# On thin ice: barriers to adoption of surveillance for patients with stage I testicular seminoma. Survey of US radiation oncologists

**DOI:** 10.1590/S1677-5538.IBJU.2017.0454

**Published:** 2018

**Authors:** Aditya Jain, Catherine Degnin, Yiyi Chen, Mike Craycraft, Arthur Hung, Jerry Jaboin, Charles R Thomas, Timur Mitin

**Affiliations:** 1 Boston University, Boston, MA, USA; 2 Oregon Health and Science University, Portland, OR, USA; 3 Testicular Cancer Society, Cincinnati, OH, USA

**Keywords:** Seminoma, Chemotherapy, Adjuvant, Radiotherapy, Health Care Surveys

## Abstract

**Purpose:**

Most men with stage I testicular seminoma are cured with surgery alone, which is a preferred strategy per national guidelines. The current pattern of practice among US radiation oncologists (ROs) is unknown.

**Materials and Methods:**

We surveyed practicing US ROs via an online questionnaire. Respondent's characteristics, self-rated knowledge, perceived patient compliance rates with observation were analyzed for association with treatment recommendations.

**Results:**

We received 353 responses from ROs, of whom 23% considered themselves experts. A vast majority (84%) recommend observation as a default strategy, however this rate drops to 3% if the patient is believed to be noncompliant. 33% of respondents believe that survival is jeopardized in case of disease recurrence, and among these respondents only 5% support observation. 22% of respondents over-estimate the likelihood of noncompliance with observation to be in the 50-80% range. Responders with a higher perceived noncompliance rate are more likely to recommend adjuvant therapy (Fisher's exact p<0.01). Only 7% of respondents recommend observation for stage IS seminoma and 45% administer adjuvant RT in patients with elevated pre-orchiectomy alpha-fetal protein levels.

**Conclusions:**

Many US ROs over-estimate the likelihood that stage I testicular seminoma patients will be noncompliant with surveillance and incorrectly believe that overall survival is jeopardized if disease recurs on surveillance. Observation is quickly dismissed for patients who are not deemed to be compliant with observation, and is generally not accepted for patients with stage IS disease. There is clearly an opportunity for improved physician education on evidence-based management of stage I testicular seminoma.

## INTRODUCTION

Testicular seminoma is the most common malignancy among young men, and it is highly sensitive to chemotherapy and radiation therapy with excellent cure rates, even in patients with metastatic disease (1). Virtually all patients with stage I testicular seminoma are cured regardless of post-orchiectomy management, and several large prospective population-based studies revealed no difference in overall survival outcomes in patients treated with adjuvant therapies or observed clinically (2, 3). Current US national guidelines recommend observation as preferred option for patients with this diagnosis (4). The most recently published National Cancer Data Base analysis revealed a dramatic increase in uptake of observation between 1998 and 2011 (5), yet the survey of practicing radiation oncologists in the United States conducted in 2010 showed an overwhelming recommendation for adjuvant RT and a very poor acceptance of observation (6). We hypothesized that over the past 6 years US radiation oncologists would have embraced observation as the first choice option for these patients, and we wanted to determine if physicians take into consideration clinical tumor features as well as concerns for poor compliance with observation protocol in making clinical recommendations. Finally, the optimal management is unclear for the small percentage of patients with stage I disease who have elevated serum markers after orchiectomy (stage IS). Using an anonymous electronic survey of practicing US radiation oncologists we have set out to examine the most contemporary patterns of practice.

## MATERIALS AND METHODS

### Survey Instrument Development

The study was approved by the Oregon Health and Science Institute (OHSU) institutional review board. The online survey was developed using the Research Electronic Data Capture (REDCap) software licensed by the Oregon Clinical and Translational Research Institute (OCTRI) for use by OHSU. The survey contained 25 questions regarding respondent demographics and treatment recommendations for various clinical scenarios. Branching logic was used to tailor the questions based on previous responses, such that most respondents were not exposed to all 25 questions. Respondent characteristics included information regarding practice setting (academic or private), years since completion of residency, and geographical location. Additionally, respondents self-rated their depth of knowledge in the field of testicular seminoma and were grouped into three categories - not comfortable with evaluating a patient with seminoma, comfortable with evaluating and treating, and expert level knowledge of the field.

### Data Collection

The data sample was collected through a fully anonymous online survey of radiation oncologists in the United States, who are members of the American Society for Radiation Oncology. Each participant was contacted and invited to complete the survey using the REDCap tool. The invitation contained instructions for participation, information regarding the study, and contact information. The first invitation was sent on September 8^th^, 2016. Participants who requested not to be contacted in the future were immediately removed from the database. The remaining respondents were contacted with a reminder email on September 18^th^, 2016, to maximize response rate. No further communication with participants ensued.

#### Statistical analysis

Respondents were characterized by years since residency completion, number of testicular seminoma patients evaluated in the past year, number of testicular seminoma patients treated with radiotherapy in the past year, practice setting, geographic region of practice, and self-rated knowledge. These six factors were analyzed for correlation with respondent treatment recommendations. Pearson's chi-square test was used to examine the correlation between characteristics and treatment questions. Cochran-Armitage analysis was used to assess trends in change of ordinal categorical values. Fisher's exact test was utilized to quantify deviation from the null hypothesis in relatively small sample sizes. A p-value <0.05 was considered statistically significant. SAS 9.4 (NY, Cary) was used for statistical analysis.

## RESULTS

### Survey Respondents

The survey was sent to 6967 email addresses, some of which could belong to the same individuals as the developed database used both personal and institutional email addresses. We received 712 undeliverable/failed automatic responses, 74 non-applicable/ineligible responses, and 354 completed responses, among which one was from a non-radiation oncologist, thus excluded from analysis. Characteristics of 353 radiation oncologists are summarized in Table-1. Most respondents (>70%) were practicing for over 10 years after completing residency training, most (73%) felt comfortable evaluating and treating patients with testicular seminoma and 23% considered themselves experts in this field. Over 30% of respondents did not see any patients with testicular seminoma over the past year and over 50% did not treat testicular seminoma with radiation therapy over the course of the year. Very few (<3%) treated more than 5 patients with testicular seminoma over the past 12 months.

**Table 1:** Characteristics of radiation oncologists who completed the survey.

		Number of respondents (%)
**Number of years after completion of residency training**		
	Currently in residency training	10 (2.83%)
	0-2	15 (4.25%)
	3-5	26 (7.37%)
	6-10	48 (13.60%)
	over 10	254 (71.95%)
**Number of testicular seminoma patients evaluated over the past 12 months**		
	0	113 (32.01%)
	<5	215 (60.91%)
	5-10	18 (5.10%)
	>10	7 (1.98%)
**Number of testicular seminoma patients treated with RT over the past 12 months**		
	0	188 (53.25%)
	<5	155 (43.91%)
	5-10	7 (1.98%)
	>10	3 (0.85%)
**Practice setting**		
	Academic Center	128 (36.26%)
	Private Practice	225 (63.74%)
**Practice region**		
	Northern	81 (22.95%)
	Pacific	62 (17.56%)
	Southern	71 (20.11%)
	Western	40 (11.33%)
	Central	85 (24.08%)
	Others/Unknown	12 (3.40%)
	Canada	2 (0.57%)
**Self-assessed depth of knowledge in the field of testicular seminoma**		
	Not comfortable evaluating patients	14 (3.97%)
	Comfortable evaluating, but not an expert	257 (72.80%)
	Expert in this field	82 (23.23%)

### Most Respondents Recommend Observation for Compliant Patients with Stage I Testicular Seminoma

When given a clinical scenario of a 20-year-old man with stage I left-sided testicular seminoma, 84% of respondents recommended observation following orchiectomy, while RT was recommended by 10% and chemotherapy by 6% (Table-2). There was a significant difference between responses from physicians in academic institutions and private practices, with RT recommendation by 3% and 13%, chemotherapy by 9% and 6% and observation by 88% and 81% of academic and private physicians, respectively (p=0.003). Respondents who recommended chemotherapy were more likely not to have seen a single testicular seminoma patient in consultation over the past 12 months, compared to respondents who recommended observation or RT (p=0.02). Respondents who did not treat a single testicular seminoma patient with RT over the past 12 months were more likely to recommend observation or chemotherapy to their patients than RT (p<0.01).

**Table 2:** Adjuvant treatment recommendations for patients with stage i testicular seminoma.

	Adjuvant Treatment Type Recommended % (N)		
	Observation	Radiation Therapy	Chemotherapy
For a young patient with small pure seminoma (N=353)	84% (296)	10% (34)	6% (23)
For a patient who is judged to be poorly compliant (N=296)	3% (21)	67% (197)	30% (89)
For a patient with pure seminoma but persistently elevated markers after orchiectomy (Stage IS) (N=353)	7% (26)	28% (100)	64% (227)

### Fear of Perceived Noncompliance with Clinical Follow-up Affects Adjuvant Treatment Recommendation

**Figure 1:**
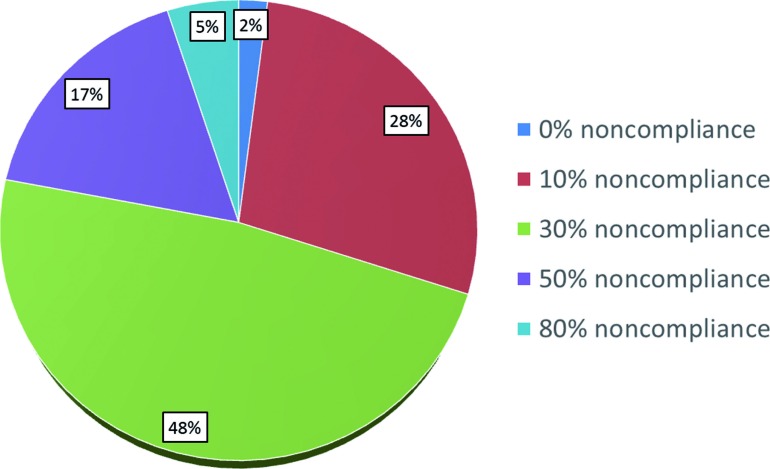
Perceived rate of noncompliance with clinical follow-up for patients with Stage I testicular seminoma among US radiation oncologists.

Among respondents, 22% estimate the rate of noncompliance with surveillance to be 50-80% (Figure-1). The fear of poor compliance dramatically affected the adjuvant treatment recommendations among our respondents, with only 3% still recommending observation, 67% recommending RT, and 30% recommending chemotherapy among those 296 respondents who initially endorsed observation (Table-2). There was a strong correlation between recommending observation in the adjuvant setting and perceiving noncompliance among men with testicular seminoma to be in the low range of 10 to 30%, whereas practitioners who believe the rate of noncompliance is over 30% in general are less likely to recommend observation (p<0.01).

### Fear of Jeopardizing Patient's Survival in Case of Disease Progression Affects Adjuvant Treatment Recommendation

Thirty three percent (n=118) of respondents believe that survival outcomes are jeopardized if a patient's testicular seminoma progresses and/or metastasizes in the absence of adjuvant therapy and strict observation protocol. Among these 118 respondents, only 5% recommend observation as an adjuvant treatment for an otherwise healthy and compliant man with a small testicular semi-noma, compared to 26% who recommend RT and 69% who recommend chemotherapy. There was a significant association between higher self-rated perceived depth of knowledge in the field of testicular seminoma and believing that survival is not jeopardized in case of testicular seminoma progression (p=0.03).

### Clinical Factors (Tumor Size, Rete Testis, History of Prior Pelvic Surgery) Influence Treatment Recommendations

Sixty percent of respondents take into consideration tumor size and rete testis invasion when they recommend adjuvant treatment (Table-3). Recent graduates were more likely not to consider these clinical factors in their decision than physicians with more years of clinical experience (p=0.008), and there was a trend towards foregoing these considerations with increased self-reported knowledge (p=0.059). Consideration of these clinical factors was not associated with practice setting or the number of testicular seminoma patients evaluated or treated with RT. 42% (149) take into consideration history of prior pelvic surgery. Among these 149 respondents, for a patient with a history of prior pelvic surgery, only 10% recommend observation, whereas 41% recommend adjuvant chemotherapy and 49% recommend adjuvant RT.

**Table 3:** Influence of clinical factors on treatment recommendation.

Clinical Factor		Number of respondents (%)
**Tumor Size and Rete Testis Invasion**		
	Affects treatment recommendation	211 (60%)
	Does not affect treatment recommendation	142 (40%)
**History of prior pelvic surgery**		
	Affects treatment recommendation	149 (42%)
	Does not affect treatment recommendation	204 (58%)

### Elevated Markers (Stage IS) Affect Treatment Recommendations

Only 7% (N=26) of respondents recommend observation for patients with elevated markers post-orchiectomy (Stage IS), whereas 28% recommend adjuvant RT and 64% recommend adjuvant chemotherapy (Table-2). Among these 26 respondents who support observation for Stage IS, 77% do not believe that disease progression or metastases jeopardize survival outcome in patients with testicular seminoma. When physicians were asked whether they would be ready to administer RT to a patient desiring this adjuvant treatment modality in the setting of pure seminoma by pathology but elevated pre-orchiectomy AFP levels, 44% of respondents supported the use of RT. There were no statistically significant associations between agreeing or refusing to offer adjuvant RT to patients with elevated AFP levels and self-described level of experience, number of seminoma patients evaluated or treated, or number of years beyond residency training. Practice setting was associated with treatment decision, with 64% of academic physicians and 51% of private practice practitioners refusing adjuvant RT with elevated AFP levels (p=0.02).

## DISCUSSION

### Adjuvant Treatment Recommendations

A recently published National Cancer Data Base analysis showed a dramatic change in management of patients with Stage I testicular seminoma between 1998 and 2011 in the US - with observation rates increased from 24% to 54%, while adjuvant RT rates decreased from 70% to 30% over these years (5). A previous survey of practicing radiation oncologists in US conducted in 2010 revealed only 20% of respondents recommending observation, with 60% recommending adjuvant RT and 3% recommending adjuvant chemotherapy. Our survey reveals contemporary patterns of care and provides basis for evaluating the practice change over the course of the last 6 years in the United States, with an overwhelming support for observation (84%) among radiation oncologists for compliant patients with no adverse features, such as large tumor size, rete testis invasion, history of prior pelvic radiation, or post-orchiectomy elevated tumor markers. This is an important victory for evidence-based practice, as several large prospective population-based studies revealed no difference in overall survival outcomes in patients treated with adjuvant therapies or observed clinically (2, 3), largely based on availability of highly efficacious salvage therapies. Moreover, a randomized trial MRC TE19/EORTC 30982 of carboplatin vs. radiation therapy with a median follow-up of over 6 years revealed no difference in relapse-free survival, and adjuvant chemotherapy resulted in a dramatic reduction in the rate of contralateral germ cell tumor development (HR 0.2) (7). For physicians concerned with disease progression in patients with stage I testicular seminoma, adjuvant chemotherapy could therefore be considered an excellent, if not superior, alternative to adjuvant RT. In fact, based on the results of a prospective population-based study in Norway and Sweden, radiation therapy has been completely abandoned as a treatment option for patients with Stage I seminoma in these countries, largely due to an increasing burden of proof of late radiation-induced secondary malignancies (8-10). Because Scandinavian oncologists are both radiation and medical oncologists by training and practice-and since diagnostic, treatment, and follow-up procedures are virtually free of charge to patients-this important healthcare decision appears to be based on clinical considerations rather than any economic incentives. In our survey radiation oncologists in private practice are more likely to recommend adjuvant RT than their colleagues in academic centers (13% vs. 3%, p=0.003), suggesting there is either less of an economic incentive in academic centers, or a better coordination of multi-disciplinary care that leads to more evidence-based patient-centered management recommendations.

### Fears that drive radiation oncologists away from observation

Unfortunately, our survey results show that observation as a preferred management option is quickly dismissed when logistical or clinical features make patients with Stage I testicular seminoma appear to be less than desirable candidates for observation. Perceived noncompliance with observation protocol is one of the main concerns among US radiation oncologists. Seventy-six percent of our respondents have correctly identified the rate of noncompliance in the general population (10-30%), as supported by published reports (11). If a patient is perceived to be noncompliant, 67% of radiation oncologists recommend RT, 30% recommend chemotherapy, and only 10% continue to recommend observation. This dramatic change in recommendation can only in part be explained by fear of jeopardizing patient survival if disease recurrence is not detected early due to poor compliance, as only 33% of our respondents hold this view. The preference for adjuvant treatments in this scenario is not evidence based, as outcomes were shown to be similar among testicular cancer patients with good and poor compliances (12, 13). Moreover, 70% of our respondents do not believe that disease progression affects patient's outcomes, as salvage treatments are highly effective, bringing survival rates even among patients with metastatic seminoma into high 90 percent rates. In a large population-based analysis of patterns of relapse in men with Stage I testicular seminoma managed with active surveillance, 99% of relapses exhibited good-risk features, and all recurrences were cured with standard therapies (14). The fear of worse outcomes is less common among self-rated experts, so physician education is necessary to alleviate this fear among providers. But there may be an entrenched teaching among US radiation oncologists based on earlier guidelines and high profile manuscripts that suggested compliance should be an important consideration in patient evaluation and management decision (15). The time may have come to re-evaluate these dogmas and update contemporary teaching materials. Likewise, clinical factors, such as tumor size and rete testis invasion, have been previously considered prognostic factors for disease relapse and suggested to be incorporated in the treatment decision regarding adjuvant treatment, based on multi-institutional data published in 2002 (16). However, this analysis was updated in 2015 and revealed that rete testis invasion was no longer an independent factor, and the tumor cut-off size could no longer be used in the prognostic model for relapse. The authors concluded that the use of risk-adapted therapy based on the model incorporating tumor size and rete testis invasion was not recommended in clinical practice (17). Among our respondents 60% still routinely base their treatment recommendations on the presence of these two clinical factors. Another 40% of respondents take into consideration the history of prior pelvic surgery. Even though one could argue that the surgical disruption of tissue planes may alter the lymphatic drainage and hence lead to an altered pattern of disease spread, there is no evidence or rational explanation for potentially increased risk of disease recurrence on observation due to prior pelvic surgery. More education is necessary to dispel fears of increased disease progression among physicians and patients and empower patients not to fear disease recurrence due to highly effective treatment options available in the salvage setting.

### Management of Stage IS Testicular Seminoma

Patients with elevated markers post-orchiectomy have Stage IS disease. This clinical scenario is not common, accounting for less than 5% of all patients with Stage I testicular seminoma based on NCDB analysis (5, 18). The best management option for these patients is unknown, since radiotherapy-based clinical trials have always ex-cluded these patients (19), as did the randomized trial of chemotherapy vs. RT (7). It is also unclear from population-based analyses which management option results in the optimal long-term outcomes for patients with Stage IS testicular seminoma (18, 20). Current NCCN guidelines recommend repeating elevated serum tumor markers, assessing with abdominal/pelvic CT scan for evaluable disease, and treating according to extent of disease at relapse, rather than offering these patients adjuvant treatment in the absence of a clearly visible disease target (4). This is in stark contrast to our survey results, which reveal that 64% of respondents recommend adjuvant chemotherapy, 28% adjuvant RT, and only 7% observation. An even less common scenario-but one that occasionally confuses both patients and physicians-is when pathology reveals pure seminoma but pre-orchiectomy alpha fetal protein (AFP) levels are elevated. Among our responders 44% were comfortable administering adjuvant RT. It is important to realize that elevated AFP is a hallmark of a non-seminoma germ cell tumor, and adjuvant RT is an inappropriate management in this clinical scenario.

### Limitations

The greatest limitation of our study is a low response rate with a sample size of 353 evaluable responses. Our findings have to be interpreted with great caution, as they may not be representative of other radiation oncologists who chose not to participate in the survey. Very few respondents evaluated 5 or more patients with testicular seminoma in the span of a year, however, almost a quarter considered themselves experts in this field. This dichotomy is likely explained by decrease in referral of these patients to radiation oncologists in the United States.

## CONCLUSIONS

There is a dramatic increase in acceptance of observation among US radiation oncologists treating patients with Stage I testicular seminoma. Our survey highlights fears and misconceptions among practicing US radiation oncologists that impede a wider adoption of observation strategy, revealing a dire need for educational outreach to minimize unnecessary treatments and reduce treatment-related toxicity among patients with Stage I testicular seminoma.

## References

[1] Horwich A, Shipley J, Huddart R (2006). Testicular germ-cell cancer. Lancet.

[2] Mortensen MS, Lauritsen J, Gundgaard MG, Agerbæk M, Holm NV, Christensen IJ (2014). A nationwide cohort study of stage I seminoma patients followed on a surveillance program. Eur Urol..

[3] Tandstad T, Smaaland R, Solberg A, Bremnes RM, Langberg CW, Laurell A (2011). Management of seminomatous testicular cancer: a binational prospective population-based study from the Swedish norwegian testicular cancer study group. J Clin Oncol..

[4] Testicular Cancer. National Comprehensive Cancer Network. v2.2018.

[5] Gray PJ, Lin CC, Sineshaw H, Paly JJ, Jemal A, Efstathiou JA (2015). Management trends in stage I testicular seminoma: Impact of race, insurance status, and treatment facility. Cancer..

[6] Arvold ND, Catalano PJ, Sweeney CJ, Hoffman KE, Nguyen PL, Balboni TA (2012). Barriers to the implementation of surveillance for stage I testicular seminoma. Int J Radiat Oncol Biol Phys..

[7] Oliver RT, Mead GM, Rustin GJ, Joffe JK, Aass N, Coleman R (2011). Randomized trial of carboplatin versus radiotherapy for stage I seminoma: mature results on relapse and contralateral testis cancer rates in MRC TE19/EORTC 30982 study (ISRCTN27163214). J Clin Oncol..

[8] van Leeuwen FE, Stiggelbout AM, van den Belt-Dusebout AW, Noyon R, Eliel MR, van Kerkhoff EH (1993). Second cancer risk following testicular cancer: a follow-up study of 1,909 patients. J Clin Oncol..

[9] Horwich A, Fossa SD, Huddart R, Dearnaley DP, Stenning S, Aresu M (2014). Second cancer risk and mortality in men treated with radiotherapy for stage I seminoma. Br J Cancer..

[10] Zagars GK, Ballo MT, Lee AK, Strom SS (2004). Mortality after cure of testicular seminoma. J Clin Oncol..

[11] Alomary I, Samant R, Gallant V (2006). Treatment of stage I seminoma: a 15-year review. Urol Oncol..

[12] Yu HY, Madison RA, Setodji CM, Saigal CS (2009). Quality of surveillance for stage I testis cancer in the community. J Clin Oncol..

[13] Ernst DS, Brasher P, Venner PM, Czaykowski P, Moore MJ, Reyno L (2005). Compliance and outcome of patients with stage 1 non-seminomatous germ cell tumors (NSGCT) managed with surveillance programs in seven Canadian centres. Can J Urol..

[14] Kollmannsberger C, Tandstad T, Bedard PL, Cohn-Cedermark G, Chung PW, Jewett MA (2015). Patterns of relapse in patients with clinical stage I testicular cancer managed with active surveillance. J Clin Oncol..

[15] Wilder RB, Buyyounouski MK, Efstathiou JA, Beard CJ (2012). Radiotherapy treatment planning for testicular seminoma. Int J Radiat Oncol Biol Phys..

[16] Warde P, Specht L, Horwich A, Oliver T, Panzarella T, Gospodarowicz M (2002). Prognostic factors for relapse in stage I seminoma managed by surveillance: a pooled analysis. J Clin Oncol..

[17] Chung P, Daugaard G, Tyldesley S, Atenafu EG, Panzarella T, Kollmannsberger C (2015). Evaluation of a prognostic model for risk of relapse in stage I seminoma surveillance. Cancer Med..

[18] Kamran SC, Seisen T, Markt SC, Preston MA, Frazier AL, Sweeney CJ (2017). Post-orchiectomy adjuvant therapy versus surveillance for stage IS testicular cancer. 2017 Genitourinary Cancers Symposium; Orlando, Florida: Journal of Clinical Oncology;.

[19] Jones WG, Fossa SD, Mead GM, Roberts JT, Sokal M, Horwich A (2005). Randomized trial of 30 versus 20 Gy in the adjuvant treatment of stage I Testicular Seminoma: a report on Medical Research Council Trial TE18, European Organisation for the Research and Treatment of Cancer Trial 30942 (ISRCTN18525328). J Clin Oncol..

[20] Ahmed KA, Wilder RB (2014). Outcomes and treatment patterns as a function of time in stage IS testicular seminoma: a population-based analysis. Cancer Epidemiol..

